# Body Size of Children and Adolescents from Sports Schools in the Municipality of Neiva-Huilla-Colombia

**DOI:** 10.3390/sports13110371

**Published:** 2025-10-27

**Authors:** José David López Laiseca, Kevin Ian Norton, Luís Miguel Massuça

**Affiliations:** 1 Faculty of Physical Education and Sport, Universidade Lusófona, 1749-024 Lisbon, Portugal; 2 Alliance for Research in Exercise, Nutrition and Activity (ARENA), Allied Health and Human Performance, University of South Australia, GPO Box 2471, Adelaide, SA 5001, Australia; 3 School of Health Sciences, University of New South Wales (UNSW), Sydney, NSW 2033, Australia; 4 Center for Sport, Physical Education, Exercise and Health (CIDEFES), Universidade Lusófona, 1749-024 Lisbon, Portugal; 5 Centre of Research, Education, Innovation and Intervention in Sport (CIFI2D), Faculty of Sport, Universidade do Porto, 4200-450 Porto, Portugal; 6 Police Research Center (ICPOL), Higher Institute of Police Sciences and Homeland Security, 1300-663 Lisbon, Portugal

**Keywords:** morphology, team sports, talent selection, young athletes, youth sports

## Abstract

Background: Accurate anthropometric data provides insight into comprehending morphological optimisation in sports. This study aims to determine the probability that a randomly selected individual from the general reference population falls within the sports-specific population. Methods: This cross-sectional study included 18,462 children and adolescents (9–14 years) from the Huila department (general population, n = 17,895) and the Neiva City Sports Schools representing athletes in team sports (sport-specific population, n = 567). Body size attributes (weight and height) were considered, and the bivariate overlap zone (BOZ) was calculated. A valid and noninvasive method was adopted to estimate the maturity offset and, complementarily, the age at peak height velocity. Results: (i) BOZ results demonstrate body size is relatively more important at younger ages compared to adolescent years in both girls and boys across all sports; (ii) differences in BOZ values are clear across different sports; (iii) basketball consistently showed higher BOZ scores in both genders indicating a relatively greater opportunity for selection in this sport based on body size alone; and (iv) BOZ values tend to be substantially lower for boys when compared to girls, particularly in the older age groups. Conclusions: Our study suggested (i) more competition for sports selection among boys, likely too much selection emphasis on body size among the youngest children, and (ii) reduced competition for sports selection among young adolescents, particularly girls.

## 1. Introduction

The literature suggests that motor skills (development and a basic assessment) are fundamental for the future of children and adolescents in terms of performance and health [1]. Nevertheless, accurate anthropometric reference data are also necessary for coaches, physicians, and other health professionals. It allows individuals to compare their values with those of their peers, providing a better understanding of the normal variation within sports functions related to physical fitness and position on the field. Additionally, normative data are vital for monitoring the growth and development of children to ensure optimal health.

Recognising athletic talent in youth divisions is essential for professional sports [2]. Countries and teams that can identify and train young athletes with potential early on have an advantage in future sports competitions [3,4]. The search for future athletes begins with young athletes who exhibit potential in a specific sport [5]. Statistical analysis can identify talented athletes and reduce the expenses associated with talent development [6]. However, it is essential to note that performance profiles during childhood may differ significantly from adulthood [7].

Research on team sports has shown that the anthropometric attributes of basketball players are crucial for their performance and the selection of skilled players [8]. The same applies to soccer, where body traits and physical abilities are significant factors for identifying talent in young players [2,9]. Additionally, understanding the anthropometric profile of volleyball players, particularly in relation to their playing position, is crucial for effective selection and success [10]. The physical fitness of athletes is a key factor in their success in nearly all sports [11]. These traits determine an athlete’s suitability for specific sports and positions and can affect performance and progression [12,13].

According to Ferioli et al. [14] and Nieman and Wentz [15], improving motor skills and morphological attributes are crucial for player development. Additionally, elite athletes tend to possess common physical fitness traits, particularly as they progress in their sport. This allows coaches and team administrators to create effective training programmes and identify promising athletes early on. Understanding the successful traits of top athletes can also lead to the creation of normative datasets, which can be used to compare results across different ages and genders and identify critical factors for individual performance development.

Considering the above, this study aims to observe, recognise, and validate the connections among different anthropometric attributes, such as body size attributes (i.e., weight and height), which are crucial factors for selecting young athletes in various sports. For instance, not being tall can be disadvantageous in sports like basketball or volleyball, as it prevents players from reaching higher or wider targets during the game. In basketball, taller players and those with powerful jumping abilities may have an edge in both defensive and offensive plays, such as blocking [16]. Hence, optimising young athletes’ sports performance is imperative to enhance their potential and excel in a particular sport.

In accordance, this research aims to analyse the body size of children and adolescents aged 9 to 14 who participate in team sports at sports schools in Neiva, Huila, Colombia, and to determine the probability that a randomly chosen individual from the reference population (Huila, Colombia) falls within the team sports population to understand the selection pressure for athletes based on body size (anthropometry) alone.

## 2. Materials and Methods

### 2.1. Study Population

**Figure 1:**
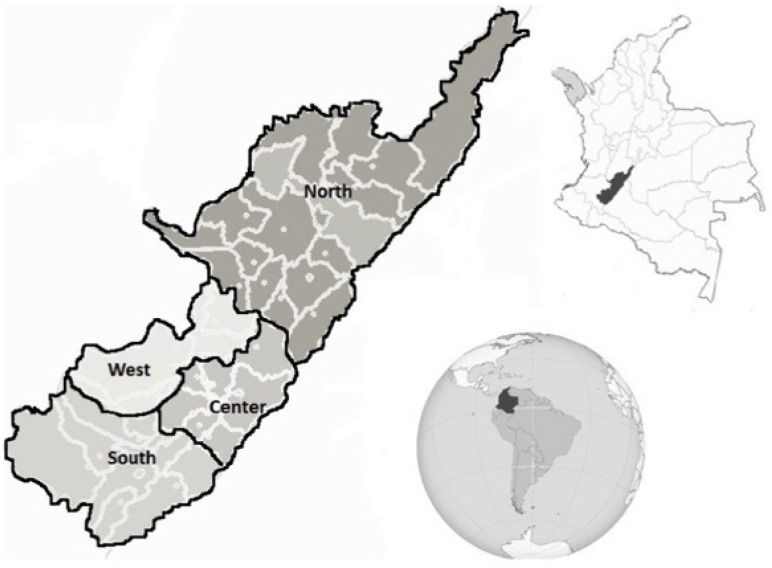
Geographic characterisation of the department of Huila—Colombia.

The Huila department is located in the southern region of Colombia and is divided into four areas: centre, north, west, and south (Figure 1). This unique department boasts a variety of climates, with temperatures varying according to altitude. The population of Huila is 1,001,476 (adjusted population as of 30 June 2005, of 1,011,418 inhabitants), with 600,801 residing in the municipal capitals and 400,675 in the rest of the Huila territory, based on the 2005 census [17]. The population comprises 97.8% mestizos, 1.2% Afro-descendants, and 1.1% indigenous ethnic groups.

Body weight and height from the reference population—department of Huila, which included 17,895 children and adolescents (aged 9 to 14 years) from the Huila department (female, n = 9898; male, n = 7997) (see López-Laiseca & Massuça [18]) and 567 athletes selected by coaches from the general Sports School’s population (team sports: basketball, soccer, futsal, and volleyball) in Neiva municipality (female, n = 257; male, n = 310) were considered in this study. Further details are provided in Table 1.

This study followed the ethical principles established by the Helsinki Declaration [19], the legal standards governing clinical research in humans, and the current Colombian laws regulating clinical research in humans (Resolution 008430/1993, Ministry of Health) [20]. Additionally, it was approved by the Research Ethics Committee of the Caribbean Foundation for Biomedical Research BIOS through Act number 0127, dated 31 July 2015. Informed consent was obtained from all participants (authorisation letter for data use registered with number 2017sal00002074-1 dated 22 February 2017).

### 2.2. Morphological Profile

Two attributes were considered to study the morphological profile, i.e., body weight (kg) and height (cm). Data from the reference population, the Department of Huila, were provided by the Departmental Health Secretariat of the Huila Government (see Ethical Statement) and have been studied in previous research (see López-Laiseca & Massuça [18]). Complementarily, the Neiva municipality sports school’s population were evaluated according to the standards established by the International Society for the Advancement of Kinanthropometry protocol [21]. An anthropometer (Anthropometric Kit Siber-Hegner Machines SA GPM) was used to measure height without shoes. A scale was used for body weight (Secca model 761 7019009, Body Mass Scale Vogel & Halke, Hamburg, Germany), allowing readings in 500 g intervals while wearing minimal light clothing.

### 2.3. Evaluation of Somatic Maturation (Maturity Offset, MO)

In addition to anthropometric data, maturation timing was assessed to better understand the differences in biological maturation between athletes and the general population. Since the rate and timing of maturation vary among individuals, it is essential to use a valid and noninvasive method to estimate the maturity offset (MO) and, complementarily, the age at peak height velocity (APHV). To this end, the sex-specific regression equations proposed by Moore et al. [22] were applied to predict the time in years to or from APHV from simple anthropometric measurements.

Male: MO (years) = −7.999994 + 0.0036124 × (age × height)(1)

Female: MO (years) = −7.709133 + 0.0042232 × (age × height)(2)

Estimating the MO allowed us to ensure a more accurate interpretation of the effect of growth and development on the probabilities of selection in sports.

### 2.4. Statistical Analysis

Descriptive (mean, M; standard deviation, SD) and inferential analyses (Welch’s *t*-tests) for weight and height comparisons between the general population and the sport subgroups at age categories were performed.

In continuation, we developed the normality graphs and the bivariate overlap zone (BOZ) statistics, using the differences between the average body weight and height of a reference population of young athletes from Neiva City compared to the Huila general population (note that BOZ is sensitive to changes in the reference distribution). The data processing was performed using the trial version of the Matlab programme (version R2023b), which calculated the necessary formulas described below.

When dealing with two related attributes (bivariate distributions), the BOZ is graphically represented as the overlap of two ‘density ellipses.’ For example, in the current study, we are dealing with the distribution of height (variable X) and weight (variable Y) in two populations. One population is the general population (males and females separately), referred to as the reference population (‘Ref’). The other population comprises a sample of athletes, referred to as the sports population (‘s’). We want to determine the probability that a randomly chosen individual from the reference population belongs to the sports population. We calculated the proportion of XY data points (and likelihood) from the general background population of children falling within the sports population distribution. This is performed using a statistic that follows a chi-square distribution: X^2^ = (1/(1 − rs)^2^)((((X − X)^2^)/(SX^2^)) − 2rs*(X − Xs)(Y − Ys)/((SXSY) + ((Y − Ys)^2^/(SY^2^))), where X and Y are the values of any XY data point from the reference population, rs is the correlation between the X and Y attributes in the sports population; Xs is the sample mean of variable X in the sports population; sXs is the estimated (or calculated with larger sports sample sizes) standard deviation of variable X in the sports population; Ys is the sample mean of variable Y in the sports population, and sYs is the estimated or calculated standard deviation of variable Y in the sports population.

The statistics for the sports population were collated from our sample of athletes. Once we generated the chi-square value associated with each XY data point, we calculated the corresponding probability (using two degrees of freedom). The average likelihood is our result and is referred to as BOZ. If the reference and sports populations have the same means and standard deviations for the X and Y variables and the same XY correlations, the BOZ will be very close to 0.5. As the differences between means and standard deviations increase, the BOZ will approach 0. The BOZ will approach one as the standard deviations of the X and Y variables in the sports population approach infinity. In this case, the sports population is infinitely extensive, and any data point from the reference population will fall arbitrarily close to the mean of the sports population. However, because sports populations are typically subsets of the reference population, and elite athletes within sports tend to resemble one another, their standard deviations are generally smaller than those of the reference population.

## 3. Results

Table 2 indicates that children in the sports population at nine years old, across all disciplines, display: (i) a higher weight than the general population, with variations ranging from 4 to 15 kg and an average standard deviation of ±7 kg, and (ii) higher height values, varying between 6.8 and 34 cm, with an average standard deviation of ±6.9 cm. Complementarily, it is observed that the sports population, especially girls (i) exhibit a weight higher by 2 to 9 kg compared to the general population, and (ii) concerning height, they show greater values ranging from 6 to 8 cm compared to the reference population.

By the age of 10, (i) a similar trend is observed in the higher weight of the sports population across all disciplines, ranging between 11 and 19 kg compared to the general population, and (ii) regarding height, there remains a superiority of 8 to 25 cm, which is more notable in soccer and futsal. Nevertheless, volleyball and futsal populations display (i) noticeable differences in weight, with higher values between 8 and 11 kg compared to the general population, and (ii) regarding height, although there is a tendency to be taller within a range of 13 to 16 cm (the differences are less notable than in weight).

At 11 years old, it is evident that the sports population (i) tends to have a higher weight, ranging between 9 and 16 kg, with fluctuations in the differences between sports, and (ii) in terms of height, the trend continues to be higher, varying between 10 and 13 cm, with variations according to the sport.

At 12 years old, (i) the higher weight in the sports population persists, fluctuating between 11 and 16 kg, and (ii) the superiority is maintained for height between 13 and 21 cm, varying according to the sport.

At 13 years old, the sports population (i) tends towards a higher weight, between 5 and 17 kg, with minor variations in some sports, and (ii) height also continues to be higher, varying between 9 and 18 cm, with differences depending on the sport (it is noted that futsal shows low standard deviations). It is also observed that the sports population (i) maintains a higher weight in a range of 4 to 7 kg compared to the reference population, and (ii) regarding height, the differences remain between 3 and 6 cm in the sports population.

At 14, the sports population (i) generally maintains a higher weight, being lower in basketball, and (ii) regarding height, it is notable that it fluctuates between 5 and 11 cm, with variations across different sports. Standard deviations are low in soccer and futsal. However, (i) notably, the sports population exhibits higher weight, with values in volleyball and futsal around 3 kg more than the reference population and similar in basketball but above soccer by 2.2 kg, and (ii) the sports population tends to be around 2 cm taller.

Complementarily, inferential analysis (Welch’s *t*-test) for weight and height comparisons between the general population and the sport subgroups at age categories are presented in Table 2.

Variability is observed in the BOZ in children’s basketball across different age groups. In boys, (i) at nine years old, 16.7% could play basketball, decreasing to 5.7% at age 10, then increasing to 13.8% at age 11, (ii) subsequently, there is a peak at 12 years old, reaching 25.7%, and (iii) this percentage decreases at 13 years old to 12.2%, then slightly rises again at 14 to 16.7%. In the case of girls, variability is also evident, i.e., (i) at nine years old, 11.4% have the chance of selection to play basketball, increasing to 6.8% at ten years; (ii) there is an increase at 11 years old, reaching 11.3%, and (iii) probability of being selected rises at 12 (11.4%) and 13 years (17.8%), remaining high at 14 years with 20.1%. These findings suggest a fluctuation in the chances of boys’ and girls’ selection for the basketball team across different age groups.

In terms of male soccer, results suggest that the chance of selection increases as children grow, peaking at 13 years, i.e., (i) there is a very low probability of being selected in the earlier ages, with 0.2% at nine years, 0.6% at 10 and 11 years, and 0.2% at 12 years, however (ii) there is a significant increase at 13 years, reaching 5.2%, slightly decreasing to 3.7% at 14 years. A greater chance of selection for the soccer team is observed for girls than for boys. Results suggest an upward trend in girls’ probability of being selected to play soccer, peaking at 12 years, i.e.,: (i) with 11.2% at nine years old, followed by a decrease to 0.4% at ten years and an increase at 11 years with 18.4%, subsequently (ii) a substantial increase occurs at 12 years, reaching 35.4%, decreasing at 13 years to 16.5%, and (iii) remaining relatively high probability of being selected to play soccer at 14 years with 20.1%.

In futsal, boys consistently have a low chance of selection across all age groups, ranging from 0.2% to 2.6%. For girls, the chance of being selected to play futsal is 6.2% at nine years old, peaking at 9.5% at 13 years old.

Regarding volleyball, boys’ chances of being selected gradually increase as they age, peaking at around 7.0% at 13 years and 8.2% at 14 years. Conversely, girls display a more marked increase starting from 12 years, reaching a peak around 13 years at 17.5%. These findings suggest a greater likelihood of girls at more advanced ages.

**Figure 2:**
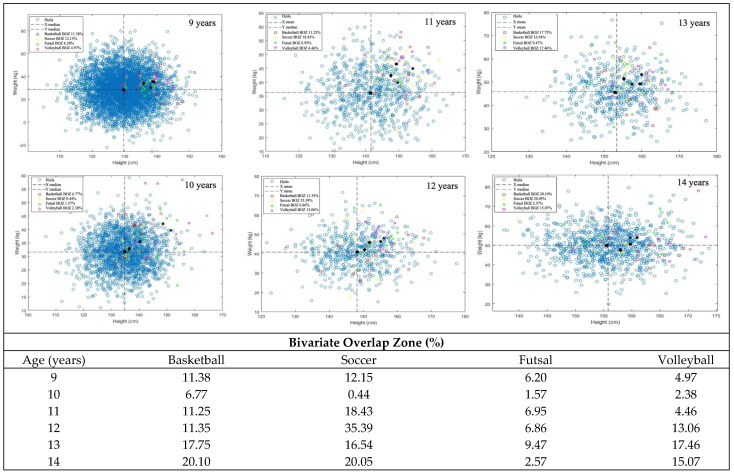
The proportion of the background population overlaps in height and weight with the sport’s height and weight of girls across age groups (9–14 years old) (Source: Authors’ original work).

**Figure 3:**
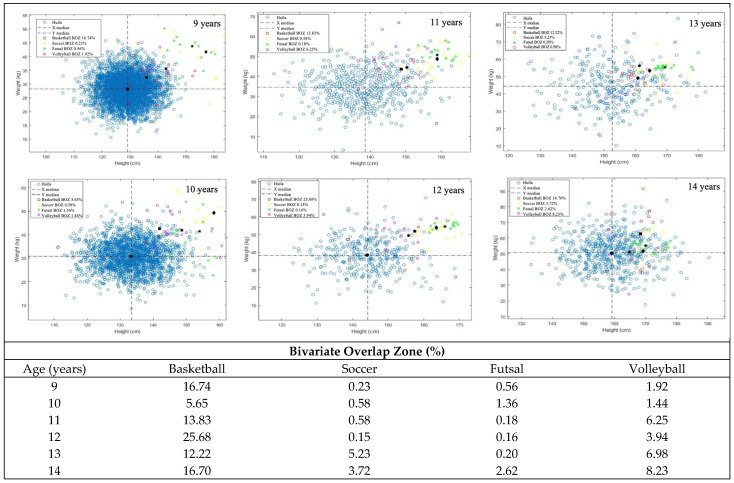
The proportion of the background population overlaps in height and weight with the sport’s height and weight of boys across age groups (9–14 years old). (Source: Authors’ original work).

Results are presented in Figure 2 (girls) and Figure 3 (boys).

**Figure 4:**
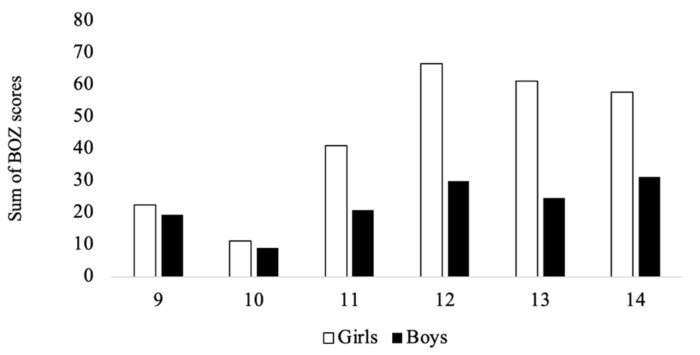
The sum of all the BOZs (basketball, futsal, soccer and volleyball) in children ages 9–14.

Variability is also observed in the sum of all BOZs (across the four sports) in children of different ages, serving as a general indicator of selection pressure for these sports. In boys, (i) at nine years old, the sum of BOZs is 19.5%, decreasing to 9.0% at age 10, then increasing to 20.8% at age 11, (ii) subsequently, there is a peak at 12 years old, reaching 29.9%, (iii) then this chance of selection decreases at 13 years old to 24.6%, and (iv) increases again to 31.3% at age 14. In girls, the proportion of the background population that overlaps in height and weight with the sports height and weight is (i) 22.6% at nine years old, decreasing to 11.2% at age 10, (ii) then increases considerably at 11 (41.1%) and 12 (66.7%) years old, (iii) subsequently, there is a slight decrease at 13 (61.2%) and 14 (57.8%) years old. Results are presented graphically in Figure 4.

It was observed that the MO of male team sports athletes only presents positive values in 14-year-old athletes (indicating that they have already surpassed the APHV). In contrast, it occurs earlier in female athletes, i.e., at 12 years (in futsal and volleyball) or 13 years (in soccer and basketball). Furthermore, were observed that: (i) male soccer players aged 9, 10 and 11, and male basketball and volleyball players aged 9 and 10, have a younger APHV than the general population of Huila (i.e., <12.9 years); and on the contrary (ii) females athletes from all team sports and of all studied ages (9 to 14), presented a later APHV than the general population of Huila (i.e., >10.5 years).

Results are presented in Table 3.

## 4. Discussion

This study analysed the body size of children and adolescents aged 9 to 14 who participated in team sports at sports schools in Neiva (Huila, Colombia) and determined the probability that a randomly chosen individual from the reference population (Huila, Colombia) fell within the team sports population to understand the selection pressure for athletes based on body size (anthropometry) alone.

The findings of this study align with previous research, indicating that anthropometric measures tend to increase with age [24]. Regarding the difference in body size attributes between boys and girls attending initiation and sports development centres compared to the general population, trained girls were found to have a greater height than their peers of the same age and gender, as observed in boys at an early age. These findings were not observed in a study by Larkin et al. [25].

Upon analysing the data from our research on sports and population, we observed that the weight and height of players of both genders in different sports showed higher values than the reference population. A notable increase in these anthropometric attributes was observed between the ages of 9 and 12 in boys, with height increases of up to 10 cm. On the other hand, in girls, increases of 6 cm were observed between the ages of 9 and 11 years. From the age of 13, boys showed increases of 5 cm; in girls, noticeable gains were maintained from 12.

In comparison with some populations between the ages of 12 and 14, it was observed that boys participating in basketball presented equivalent or lower body sizes compared to populations in Mexico [26], Brazil [27,28], and Bosnia-Herzegovina [29]. At the age of 13, they also exhibited lower measurements in mass and height compared to Mexico [26] and Bosnia-Herzegovina [29]. Involving height, soccer showed similarities with studies conducted in Chile [30] and Cuba [31], placing the above studies conducted in Brazil [32]. In the case of volleyball, at the age of 12, they showed measurements higher than Brazil [33] in both variables. However, at 13 years old, they were below the average height in Ecuador [34]. On the other hand, in futsal, higher values were observed in both variables compared to a study in Brazil [35].

For girls, similar or slightly lower measurements in body size attributes were recorded compared to research conducted in Brazil on soccer [36], basketball in Colombia [37], futsal in Brazil [35], and volleyball in Venezuela [38], with slightly higher heights in Brazil [33]. The data collected in the systematic review by López-Laiseca and Massuça [39] showed higher measurements in most age groups, particularly among boys participating in various sports, with a significant increase in weight and height between the ages of 9 and 12.

The development of body size in different sports notably increased between the ages of 9 and 12 in boys, showing asynchronous development, as described in Dolo et al. [31], while in girls, significant increases were observed between the ages of 9 and 10, which continued to align with uniform growth [18]. Additionally, girls experienced peak height velocity earlier than boys [23].

This study also highlights that the most significant growth in weight and height for girls and boys, as well as the reference population, occurs between the ages of 10 and 12 years, which aligns with previous research indicating that height growth begins around 12 years [40]. That accelerated growth may start even earlier, about 10.7 years [24]. The observed differences can be attributed to age or discrepancies in the biological maturity of players [41]. Players of the same chronological age can be ahead, on time, or behind their chronological age [42].

The complementary analysis of the athletes’ MO and APHV (Table 3) confirmed these differences in maturation. On average, boys and girls participating in the analysed team sports showed a negative MO at most ages (boys, 9 to 13 years; girls, 9 to 12 years), indicating that many had not reached their pubertal growth spurt. Moreover, (i) male soccer players aged 9, 10 and 11, and male basketball and volleyball players aged 9 and 10, have a younger APHV than the general population of Huilla [23] (i.e., <12.9 years); and on the contrary (ii) females athletes from all team sports and of all studied ages (9 to 14), presented a later APHV than the general population of Huilla [23] (i.e., >10.5 years).

This suggests that the larger body size observed in male athletes compared to the general population depends, at younger ages (9 and 10 years), on their maturation stage, i.e., suggesting that selection pressure for body size is greater in these sports at an early stage of development. However, in females, APHV was consistently later than that reported for the general population of Huila, which could contribute to the higher BOZ values in particular team sports and ages.

Complementarily, the bivariate overlap zone (BOZ) technique reveals the proportion of the background population that overlaps in height and weight with the sports height and weight of girls across age groups (9–14 years old), ranging from 0.4% to 35.4%. Across all girls’ sports: (i) the BOZ scores increase as children progress through adolescence, suggesting that body size is less critical as girls age and that other things such as skill level, dedication, and interest probably play an ever-increasing role in girls’ involvement, and (ii) at age 10, there are the lowest BOZ scores, suggesting that early matures are selected because they are bigger and probably faster and stronger as a consequence of puberty and leaving their peers behind. For example, 9% or greater BOZ scores occur in all four sports (up to 35%). These high values reinforce the many other attributes required to make team selections across all age groups in the shown sports.

The BOZ scores are typically lower for boys than girls (Figure 4). This means that selection is more challenging for boys than for girls across all age groups. In fact, across the boys’ sports, the BOZ for soccer is by far the smallest, indicating that it is very competitive to get selected (with such a low probability) between the ages of 9 and 12. Surprisingly, however, it increases as boys age up to 14 years old. Again, it likely highlights other characteristics that become relatively more important, such as tactical knowledge, skills, and motivation, compared to the younger years, where body size alone seems to be more critical.

Generally, male BOZ values vary from 0.2% to 25.7% in relation to the presence of the reference population in the sports population. However, only a relatively narrow opportunity for sports selection, based solely on body size (i.e., height and weight), is demonstrated. The BOZ is rarely above 20% but usually well below 10% of children having the necessary weight and height combination to be selected for all sports studied. There does not appear to be a pattern in the BOZ as children age in any of the sports (i.e., it rises or falls randomly), and this is likely due to a combination of “interest” among children, as well as the advantage that children have in sport (particularly at a young age) if they are bigger than other children. Moreover, in some cases, the BOZ is very small at younger ages and then becomes somewhat larger in older age groups, indicating that larger children tend to be selected in most sports before smaller children at the same age. In short, this translates into an open upper limit optimisation (i.e., “the bigger the better”).

Given the above, it seems pertinent to highlight that this study stands out for two fundamental aspects. Firstly, it addressed a significant sample of children and adolescents from sports schools specialising in training and sports development, with emphasis on basketball, soccer, futsal, and volleyball, of both genders, aged between 9 and 14 years. Since BOZ is sensitive to changes in the reference distribution, the trends observed across certain age and sex groups should be interpreted with caution. For example, the smaller groups had a representative sample of just over 300. Secondly, it examined the differences in anthropometric attributes (weight and height) between populations participating in state sports programmes and the general population at early ages.

Furthermore, it is also essential to highlight that this study pioneered the use of this technique in youth, unlike previous studies conducted by Norton and Olds [43] and Fontana et al. [44], which focused on elite sports populations and adults. Significant trends were observed regarding the representation of the reference population in various sports and age groups, particularly among minors, using the BOZ in accordance with the methods of Norton and his colleagues [13,43]. These findings underscore the importance of considering the unique anatomical composition of the reference population in each sport and age group. This knowledge can be instrumental in selecting and developing athletes in their respective disciplines and emphasises the importance of considering morphological optimisation during the youth development process in sports.

These findings are relevant to understanding the relationship between age, body size, and athletic performance, which could have significant implications for selection and development in youth sports, particularly during unpredictable stages of growth and development. Furthermore, the MO and APHV values obtained in this study can be used as complementary tools to the BOZ to better understand selection processes, allowing coaches and sports professionals to identify athletes with potential who have not yet reached their pubertal growth spurt and avoid selection biases based solely on early body size. Therefore, it is recommended that sports institutions continuously monitor anthropometric and maturation data over time, ensuring that planning and training are tailored to the individual’s stage of development, rather than solely focusing on visible physical characteristics.

Finally, it seems relevant to emphasise that (i) the study’s recruitment is based on data from the 2005 census [17], and this temporal gap may represent a critical limitation of the study, particularly in terms of demographic changes and population dynamics; and (ii) BOZ is sensitive to changes in the reference distribution and trends observed across age and sex groups are informative but not prescriptive. In general, they should be interpreted with caution. Further studies and in-depth analyses are recommended to explore the observed differences and better understand how sports practice affects the growth and development of participants in relation to the BOZ.

## 5. Conclusions

- The probability of a randomly selected individual from the general population being part of the athlete population was investigated using anthropometric variables such as weight and height.
- Valuable and updated information on the anthropometric variables of Neiva’s children, adolescents, and teenagers participating in sports schools was provided.
- Differences in weight and height were found between the analysed sports and the general population, highlighting the importance of considering morphological attributes in sports.
- Results suggested more competition for team selection among boys, likely too much selection emphasis on body size among the youngest children, and reduced competition for sports selection among young adolescents, particularly among girls, which shed light on several areas of children’s sports participation, including how selection pressures differ between genders and among sports, and how they change as children age.
- Analysis of the maturity offset (MO) and the age at peak height velocity (APHV) revealed that the maturation status of athletes varies by discipline and differs from that of the general population, influencing their likelihood of being selected for team sports. Therefore, it is crucial to consider biological maturation to understand variations in the BOZ, avoid selection bias, and promote the identification and development of talent, regardless of the individual’s rate of maturation.
- Finally, the study findings can aid coaches and sports professionals in selecting and training emerging talents, as well as in designing specific training programmes and strategies for each sports modality.

## Figures and Tables

**Table 1:** Distribution of the sample (n) according to age and gender.

Age (Years)	Huila ^1^		Selected Athletes from the Sports School’s Population in Neiva Municipality							
			Basketball		Soccer		Futsal		Volleyball	
	Female	Male	Female	Male	Female	Male	Female	Male	Female	Male
9	5248	4128	12	10	12	15	10	15	10	12
10	1989	2067	12	10	12	15	10	15	10	12
11	715	578	12	10	12	15	10	15	10	12
12	549	346	12	10	12	15	10	15	10	12
13	484	307	12	10	12	15	10	15	10	12
14	913	571	10	10	7	15	10	15	10	10
Total	9898	7997	70	60	67	90	60	90	60	70
	17,895		130		157		150		130	
	18,462									

^1^ reference population (López-Laiseca & Massuça [18]).

**Table 2:** Mean (M) and standard deviation (SD) of the regional (Huila department) and sports sample (Neiva City Sports School) by age and gender.

Age (Years)	Male			Female		
	n	Weight (kg)	Height (cm)	n	Weight (kg)	Height (cm)
		M ± SD	M± SD		M± SD	M± SD
	Huilla (n = 7997)			Huilla (n = 9901)		
9	4128	28.10 ± 4.99	129.10 ± 6.19	5248	28.43 ± 14.40	129.71 ± 6.64
10	2067	30.85 ± 6.29	133.21 ± 7.25	1989	31.72 ± 6.74	134.70 ± 7.95
11	578	34.30 ± 7.95	137.91 ± 8.32	715	36.09 ± 7.89	141.54 ± 8.51
12	346	38.48 ± 8.99	144.07 ± 8.83	549	40.99 ± 8.55	148.29 ± 8.27
13	307	44.25 ± 11.08	151.42 ± 10.41	487	45.54 ± 8.72	152.92 ± 7.67
14	571	50.41 ± 10.71	159.18 ± 9.02	913	49.82 ± 8.58	155.57 ± 6.29
	Basketball (n = 60)			Basketball (n = 70)		
9	10	32.40 ± 7.42	135.90 ± 4.65 **	12	35.35 ± 9.21 *	138.83 ± 5.97 ***
10	10	42.56 ± 7.70 ***	141.90 ± 5.24 ***	12	35.51 ± 7.79	140.16 ± 6.46 *
11	10	43.72 ± 10.25 *	148.80 ± 8.09 **	12	46.50 ± 13.66 *	149.25 ± 5.52 ***
12	10	51.96 ± 18.78 *	157.40 ± 16.70 *	12	45.79 ± 11.13	151.83 ± 8.82
13	10	49.00 ± 11.07	160.80 ± 6.21 ***	12	49.05 ± 8.84	159.58 ± 5.14 ***
14	10	62.66 ± 18.98	168.40 ± 8.58 **	10	50.62 ± 11.60	159.70 ± 6.29
	Soccer (n = 90)			Soccer (n = 67)		
9	15	41.65 ± 10.68 ***	157.06 ± 7.63 ***	12	34.03 ± 5.61 **	135.83 ± 5.35 **
10	15	49.31 ± 7.36 ***	158.33 ± 6.47 ***	12	32.83 ± 4.01	136.41 ± 0.67 ***
11	15	48.65 ± 6.92 ***	158.86 ± 3.98 ***	12	42.30 ± 8.25 *	147.58 ± 9.08 *
12	15	53.93 ± 3.90 ***	163.53 ± 3.48 ***	12	42.04 ± 12.06	150.41 ± 6.14
13	15	53.26 ± 6.48 ***	164.53 ± 5.08 ***	12	51.39 ± 9.81	155.25 ± 3.36 *
14	15	51.73 ± 9.06	169.20 ± 3.10 ***	7	47.61 ± 6.88	158.00 ± 9.80
	Futsal (n = 90)			Futsal (n = 60)		
9	15	43.80 ± 6.10 ***	152.13 ± 7.14 ***	10	30.41 ± 6.10	138.00 ± 6.15 **
10	15	41.53 ± 10.31 **	154.00 ± 7.34 ***	10	39.71 ± 8.65 *	151.40 ± 4.79 ***
11	15	50.73 ± 3.13 ***	158.80 ± 3.61 ***	10	39.80 ± 6.76	149.70 ± 3.65 ***
12	15	54.53 ± 2.23 ***	165.93 ± 3.10 ***	10	47.89 ± 5.83 **	156.10 ± 3.84 ***
13	15	55.27 ± 0.96 ***	169.33 ± 2.47 ***	10	49.00 ± 9.26	157.40 ± 5.95 *
14	15	55.15 ± 3.13 ***	170.07 ± 3.65 ***	10	53.80 ± 6.49	159.70 ± 6.46
	Volleyball (n = 70)			Volleyball (n = 60)		
9	12	35.60 ± 4.59 ***	142.80 ± 8.28 ***	10	35.58 ± 3.87 ***	138.58 ± 5.32 ***
10	12	41.87 ± 4.88 ***	148.70 ± 8.20 ***	10	42.13 ± 10.30 *	148.58 ± 8.22 ***
11	12	44.39 ± 8.40 **	150.30 ± 6.73 ***	10	44.88 ± 8.37 **	154.25 ± 5.83 ***
12	12	49.48 ± 9.40 **	155.70 ± 7.57 ***	10	46.22 ± 6.73 *	155.16 ± 5.10 **
13	12	56.24 ± 13.15 **	161.30 ± 6.76 ***	10	53.20 ± 7.73 *	160.00 ± 7.16 *
14	10	51.21 ± 10.49	164.80 ± 6.51 *	10	53.96 ± 12.51	161.00 ± 6.31 *

Key. Welch’s *t*-test for weight and height comparisons between the general population and the sport subgroups at age categories: *, *p* < 0.05; **, *p* < 0.001; ***, *p* < 0.001.

**Table 3:** Overall averages of maturity offset (MO) and age at peak height growth velocity (APHV) by sport and gender, as well as Huila APHV reference values.

Team Sports	Age (Years)	Male			Female		
		Maturity Offset (MO) (Years)	APHV (Years)	APHV Población Huila [23] (Years)	Maturity Offset (MO) (Years)	APHV (Years)	APHV Huila Reference [23] (Years)
Futsal	9	−3.44	12.05	12.9	−2.56	11.56	10.5
	10	−2.49	12.49		−1.30	11.30	
	11	−1.68	12.68		−0.77	11.77	
	12	−0.80	12.80		0.19	11.80	
	13	−0.02	13.02		0.93	12.06	
	14	0.61	13.39		1.71	12.29	
Soccer	9	−2.88	11.88		−2.55	11.55	
	10	−2.32	12.32		−2.00	12.00	
	11	−1.75	12.75		−0.91	11.91	
	12	−0.92	12.92		−0.08	12.08	
	13	−0.29	13.29		0.80	12.20	
	14	0.57	13.43		1.63	12.37	
Basketball	9	−3.53	12.53		−2.43	11.43	
	10	−2.87	12.87		−1.37	11.79	
	11	−2.26	13.26		- 0.92	11.92	
	12	−1.18	13.18		0.00	12.00	
	13	−0.44	13.44		1.08	11.92	
	14	0.52	13.48		1.73	12.27	
Volleyball	9	−3.34	12.34		−2.44	11.44	
	10	−2.04	12.04		−0.95	10.95	
	11	−2.13	13.13		−0.54	11.54	
	12	−1.25	13.25		0.16	11.84	
	13	−0.42	13.42		1.08	11.92	
	14	0.39	13.61		1.81	12.19	

## Data Availability

The data presented in this study are available on request from the corresponding author. The data are not publicly available due to privacy or ethical restrictions.
